# Research Progress in Biodiversity and Human Well-Being, Based on CiteSpace

**DOI:** 10.3390/biology13121020

**Published:** 2024-12-06

**Authors:** Sunbowen Zhang, Linsheng Wen, Aifang Weng, Dongliang Cheng, Baoyin Li

**Affiliations:** 1 School of Geographical Sciences, Fujian Normal University, Fuzhou 350117, China; qbx20240170@yjs.fjnu.edu.cn (S.Z.); qbx20220143@yjs.fjnu.edu.cn (L.W.); qbx20230150@yjs.fjnu.edu.cn (A.W.); 2 State Key Laboratory for Subtropical Mountain Ecology of the Ministry of Science and Technology and Fujian Province, Fujian Normal University, Fuzhou 350117, China

**Keywords:** biodiversity, human well-being, bibliometrics, knowledge graph, nature-based solutions (NbS)

## Abstract

Biodiversity is crucial for human well-being and economic prosperity, representing a significant factor in constructing a global community of life on Earth. This study focuses on 4453 articles indexed in the Web of Science Core Collection, employing the visualization analysis software Citespace 6.2.R7 for bibliometric analysis of research related to biodiversity and human well-being. The findings indicate the following: (1) there is a steady increase in publications on biodiversity and human well-being, covering 167 disciplinary fields, indicating a broad research scope; (2) the literature spans 168 countries and regions, with major contributions from the United States, England, Germany, Australia, and China; (3) COSTANZA R is the most cited author in this field, with *Science* being the most recognized journal; and (4) current research hot topics include ecosystem services, genetic diversity, marine protected areas, nature-based solutions (NbS), and green spaces, while climate change, urban green space management, and national park system development are identified as important emerging directions. This study, grounded in significant global literature, aims to enrich the breadth and depth of research on biodiversity and human well-being, offering feasibility references for constructing theoretical frameworks in biodiversity studies and providing theoretical guidance for promoting modern harmonious coexistence between humans and nature.

## Simple Summary

Biodiversity is crucial to human well-being and economic prosperity. This study focuses on the literature related to biodiversity and human well-being, employing bibliometric analysis methods to trace the evolution of research hotspots from 1997 to 2024, thereby clarifying the frontiers and developmental trends in this field. The results indicate that strengthening international cooperation in relevant research, focusing on ecosystem diversity, species diversity, and genetic diversity, and engaging in national park protection, climate change response, and urban green space management are essential. This study aims to provide a scientific basis for conducting research on biodiversity and human well-being, thereby offering theoretical support for advancing the harmonious coexistence of humans and nature.

## 1. Introduction

Biodiversity is capable of maintaining ecosystem functions, thereby meeting both spiritual and material human needs, and forms the cornerstone of human health and well-being and the construction of a global community of life on Earth [1]. In 1992, the Convention on Biological Diversity of the United Nations was promulgated, taking the first step towards global biodiversity conservation. Since then, various governments have subsequently enacted corresponding laws and regulations. For instance, the Chinese government has introduced policies such as the “China National Biodiversity Conservation Strategy and Action Plan (2011–2030)”, with efforts to enhance the effectiveness of biodiversity conservation. Although the level of government concern for biodiversity conservation has been increasing day by day, humanity’s destruction of biodiversity has not been effectively curbed. According to the “Living Planet Report 2020” released by the World Wildlife Fund (WWF), from 1970 to the present, due to human activities, the population of animal species has declined on average by 68%. Efforts towards biodiversity conservation remain a long and arduous task.

As a crucial guarantee for human survival and development, biodiversity has seen its related research shift from the initial studies on its essence and value ever since its concept was first proposed by Raymond in 1968 [2]. Focus has since moved towards examining the impacts of biodiversity on ecosystem services [3]. For example, Bradley J. Cardinale et al. discussed how the loss of biodiversity will change the functioning of ecosystems and their ability to provide the goods and services that societies need to thrive [4]. Productivity is also the focus of current research [5]. For example, Marquard put forward the conclusion that biodiversity is positively correlated with productivity due to an increase in plant density [6]. At the same time, environmental factors are particularly important for the study of biodiversity [7]. For example, Garcia et al. proposed that environmental factors such as temperature would change the relationship between biodiversity and ecosystem function [8]. Finally, population relations have an important impact on biodiversity [9]. For example, Li et al. showed that nutrient enrichment and species richness would directly affect the stability of populations and communities [10]. More specifically, the “UN Biodiversity 2020 Goals” explicitly emphasize the strategic objective of “enhancing the benefits of biodiversity and ecosystem services”, proposing to provide ecosystem services beneficial to health, livelihood, and well-being. This provides a realistic guide for scientific research directions for the coming period, and the enhancement of public welfare through biodiversity has become an urgent topic of importance [11].

Conceptualizing the link between biodiversity and human well-being can enhance understanding of biodiversity in scientific and policy domains, which is practically significant for implementing ecological environmental protection and sustainable development. However, with the increase in research and the expansion of the field, there are significant differences in the scientific issues emphasized by different studies. Traditional literature review methods can no longer fully reflect the research hotspots and trends of biodiversity and human well-being. Given that bibliometric analysis, based on co-citation analysis theory and pathfinding network algorithms, can reveal the knowledge structure of the research through data mining and visually express the evolution process of knowledge groups [12], this study adopts a bibliometric analysis method, using Citespace software to visually process the knowledge structure, research hotspots, and trends in this field. The study aims to address the following four questions: (1) to clarify the number of representative publications in the field; (2) to identify the distribution and co-citation status of authors and research institutions; (3) to identify the research hotspots in the field, and trace the evolution of research hotspots regarding biodiversity and human well-being from 1997 to 2024; and (4) to identify the research frontiers and development trends in the field. This study is expected to provide a scientific basis for future research on biodiversity and human well-being, thereby contributing to advancing the harmonious development of humans and nature.

## 2. Materials and Methods

**Figure 1:**
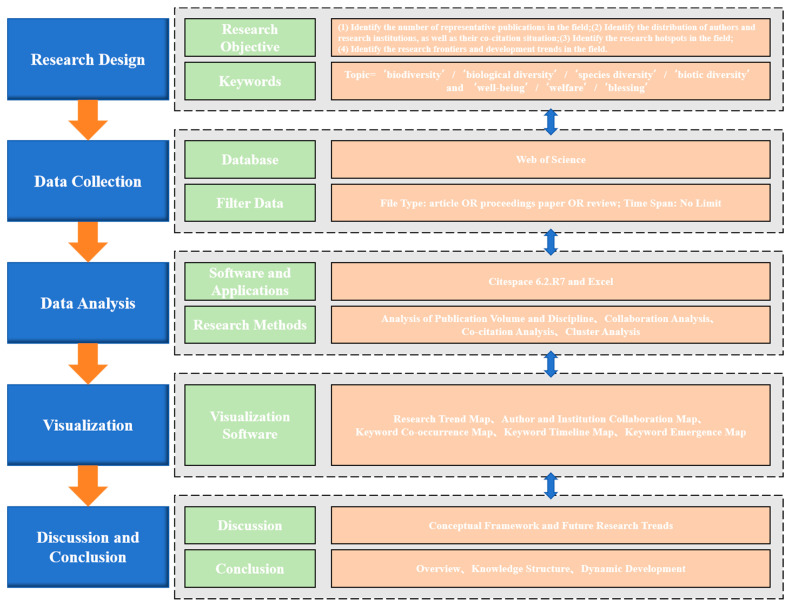
Research Framework.

As shown in Figure 1, this study is based on the Web of Science core collection database and adopts advanced retrieval methods: “Topic = ‘biodiversity’/‘biological diversity’/‘species diversity’/‘biotic diversity’ and ‘well-being’/‘welfare’/‘blessing’” are the subjects selected for the search terms. The search was conducted on 19 April 2024, and a total of 4453 academic papers on biodiversity and human well-being, published between 1997 and 2024, were included in the Web of Science Core Collection. Then, Citespace 6.2.R7 software was used to conduct quantitative statistical and visual analysis on the number of published documents, subject analysis, cooperation analysis, co-citation analysis, cluster analysis, and other aspects of relevant literature [13]. These can be divided into the following four types: (1) The number of documents and the subject analysis. A trend chart of publication volume and a subject distribution map were used to understand the inter-annual change in the number of published articles on biodiversity and human well-being and the subject of the articles, and the research trend was analyzed. (2) Cooperation analysis. Through the analysis of the cooperation network of researchers (research institutions and the country or region in which they are located), the cooperative relationship can be understood [14]. (3) Co-citation analysis. We used a citation analysis graph of the literature, authors, and journals to explore the development and evolution of the research. (4) Cluster analysis. A key words co-occurrence map was used to analyze the main viewpoints of this topic, and the characteristics and hot spots of the research were explored through the analysis of a keyword time map and an emergence map.

## 3. Results

### 3.1. Publication Volume and Fundings

**Figure 2:**
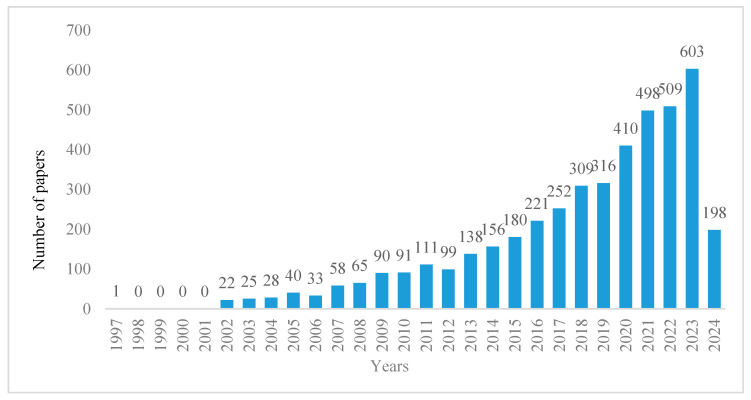
Research trend.

Through the analysis of the annual publication volume and development trend of the relevant literature, the trends in international development and attention in the research field related to biodiversity and human well-being can be reflected [13]. As shown in Figure 2, studies related to biodiversity and human well-being mainly focus on the period of 1997–2024, with an overall upward trend. The research trend can be roughly divided into the following three stages: (1) The period 1997–2006 was the initial stage, with a total of 149 papers published. With the United Nations Millennium Ecosystem Assessment project being proposed, the research on biodiversity and human well-being is entering the field of view of scholars around the world, and preliminary exploration has begun [15]. (2) The period from 2007 to 2014 was a slow development stage, with a cumulative number of 808 papers. At this stage, the number of articles increased steadily, and the research intensity remained stable. (3) Since 2015, the research has been in a stage of rapid growth, with a total of 3496 papers. During this period, the number of published papers increased rapidly, and the research efforts in biodiversity and human well-being improved significantly. In general, the focus of scholars worldwide is on research into biodiversity and human well-being, with growing intensity. More and more research findings are bridging academic studies with practical applications.

Additionally, research related to biodiversity and human well-being has received support from various funding sources, with a broad scope of funding. During the period from 1997 to 2024, the top five funders (as shown in Table 1) include the National Natural Science Foundation of China (NSFC), which provided the most funding, with 30 grants. Following this, the European Union (EU) and World Animal Protection also made significant contributions, indicating that countries around the world continue to increase their emphasis on research related to biodiversity and human well-being, ensuring that relevant studies receive adequate financial support.

### 3.2. Disciplinary Distribution

**Figure 3:**
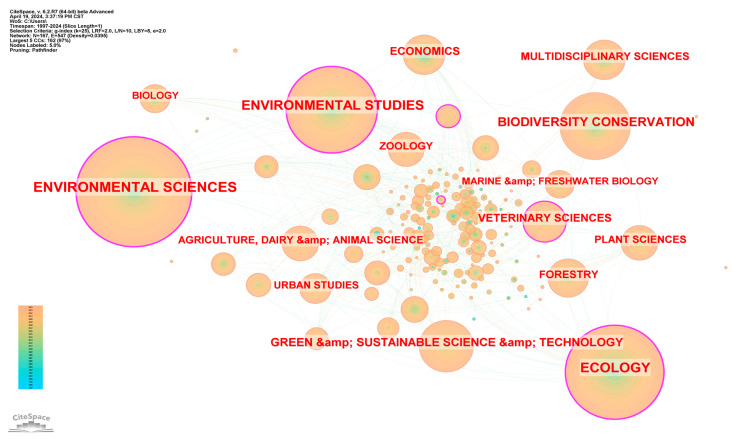
Knowledge map of disciplinary types from 1997 to 2024.

The research on biodiversity and human well-being is wide-ranging, involving a total of 167 subject areas. From 1997 to 2024, among the top 10 academic categories (as shown in Figure 3), the number of published papers in environmental science, ecology, and environmental research each exceeded 900. This indicates that the focus of research on biodiversity and human well-being is still the ecological environment. At the same time, according to the year of the first publication, plant science, pharmacology and pharmacy, chemistry, etc., carried out research on biodiversity and human well-being earlier. Since 2002, many disciplines such as economics, forestry, and urban studies have gradually come to play an important role. It can be seen that this research has gradually shifted, from early research in the field of plant science and ecological environment, to the interdisciplinary research of social science, natural science, and medicine. We suggest that with the development of human society and advances in technology, humans are increasingly recognizing that biodiversity is not only the foundation of ecosystem stability and function, but also directly or indirectly impacts human health, economic development, and social well-being. Furthermore, reductions in biodiversity may lead to increased risks of disease transmission and reduced medicinal resources, affecting public health. Therefore, interdisciplinary research bridging social sciences, natural sciences, and medicine has become the current research trend.

### 3.3. Research Countries and Institutions

**Figure 4:**
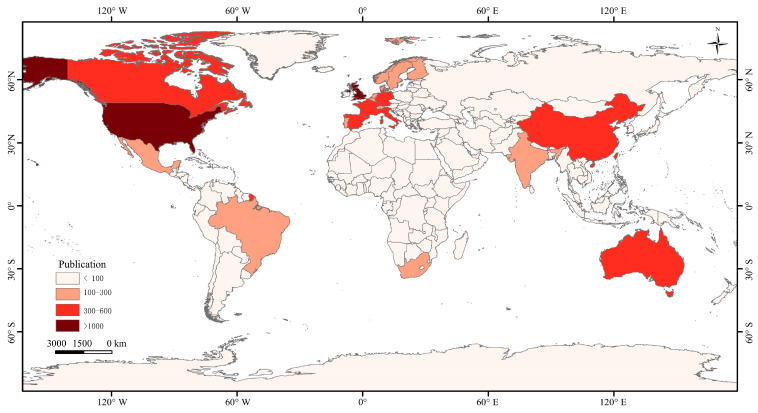
Global distribution of publication volume.

The papers on biodiversity and human well-being mainly cover 168 countries or regions. As shown in Figure 4, the largest number of publications was in the United States (1079), followed by the United Kingdom (1011), Germany (509), Australia (464), and China (449). From the perspective of national publication, the number of papers published by American scholars clearly dominates. Furthermore, as shown in Table 2, the Centre National de la Recherche Scientifique in France (CNRS) has the highest number of publications (157) from institutions, followed by the Chinese Academy of Sciences in China (132 articles), and France INRAE in France (106 articles). Although the number of papers published by American scholars is large, the papers published by their affiliated research institutions are scattered, resulting in the University of California System in the United States ranking only fourth in the world (104 papers).

**Figure 5:**
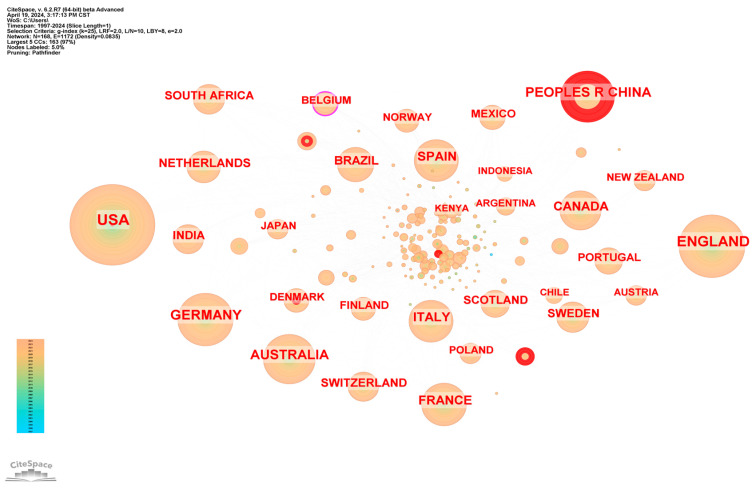
Knowledge map of cooperation network of countries (or regions) from 1997 to 2024.

The intermediary centrality of a node can reflect the influence of the node in the cooperative network [16]. As shown in Figure 5, among the countries that have produced studies on biodiversity and human well-being, Belgium has the highest intermediate centrality (0.12), followed by Scotland (0.09), Australia (0.06), Italy (0.06), and Switzerland (0.06). This shows that countries or regions such as Belgium, Scotland, Italy, and Switzerland maintain close cooperation in international cooperation, which has an important impact on research and development. The United States, England, Germany, and China were among the top five countries in the world for published papers, but their intermediate centrality did not exceed 0.05. This suggests that they have insufficient influence on the study of biodiversity and human well-being, and there is a need for more international collaborations.

### 3.4. Author and Journal Co-Citation Analysis

Co-citation refers to the co-citation relationship formed when two or more studies are cited at the same time, and the co-citation frequency can reflect the knowledge base of this research field [17]. Therefore, this study conducted co-citation analysis, using a software, to explore the ranking of authors and journals with different influences.

#### 3.4.1. Author Co-Citation Analysis

**Figure 6:**
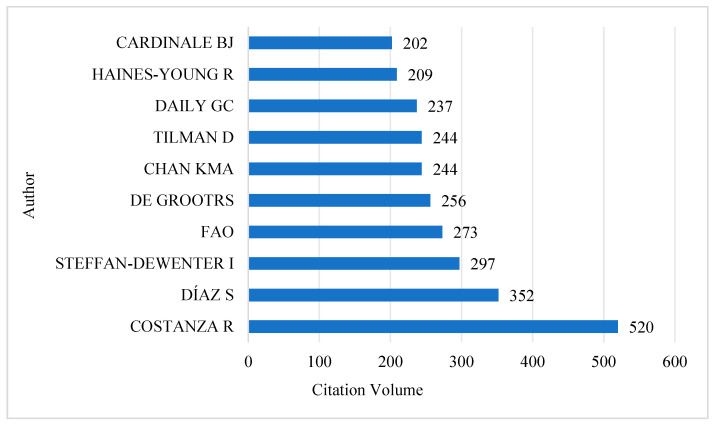
Top 10 Authors by citations from 1997 to 2024.

As shown in Figure 6, among the current studies, the author with the highest number of citations is COSTANZA R from the Australian National University, with a cumulative number of 520 citations. This makes him the most cited scholar in the field, and one of its most representative figures. Following him is DÍAZ S. from Universidad Nacional de Córdoba in Argentina, whose papers have been cited 352 times. Next is STEFFAN-DEWENTER I. from the University of Glasgow in the UK, whose papers have been cited 297 times.

#### 3.4.2. Journal Co-Citation Analysis

As shown in Table 3, *Science* is the most-cited journal, with a total of 2145 citations. In the past three years, the impact factors of *Science* were 63.7 in 2021, 56.9 in 2022, and 44.7 in 2023. Following *Science*, the next most-cited journals are *Nature*, *PNAS*, *Plos One*, and *Biological Conservation*, with citation counts of 1848, 1838, 1637, and 1471, respectively. The research further indicates that *Science*, *Nature*, and *PNAS* remain the most authoritative journals in biodiversity and human well-being research, serving as important reference literature for scholars worldwide.

### 3.5. Research Content and Frontier Progress

#### 3.5.1. Keyword Co-Occurrence Analysis

Keywords are professional terms that reflect the core views of an article, and the co-occurrence analysis of keywords can effectively illustrate the research hotspots in related fields [18]. Therefore, this study imported relevant literature data into Citespace 6.2.R7 software, and set the time span as 1997–2024, time slice as 1 year, node type as keywords, and other parameters as default values, to obtain a keyword co-occurrence map with 762 nodes, 3361 connections, and 0.0116 density. In addition, the nodes in the graph, the keyword size of labeled nodes, and the number of connections between nodes represent the keyword occurrence frequency, centrality intensity, and co-occurrence relationship, respectively.

**Figure 7:**
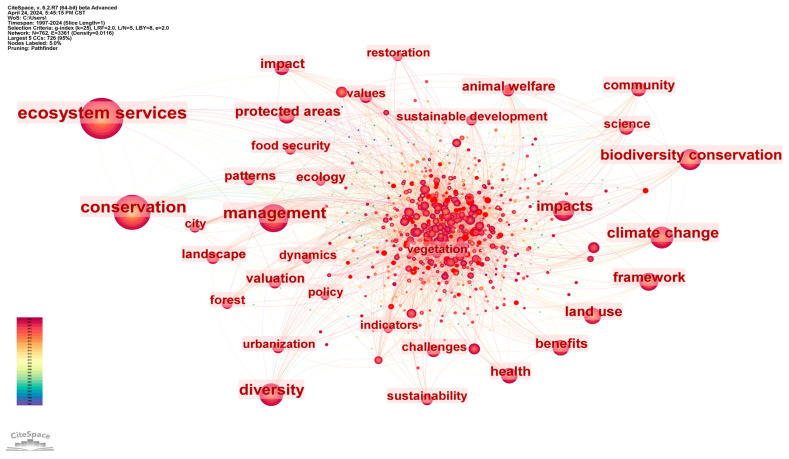
Knowledge map of keyword co-occurrence network, from 1997 to 2024.

The analysis combines overlapping keywords while removing search terms. As shown in Figure 7, the five keywords with the highest frequency are ecosystem services, conservation, management, diversity, and climate change. The five keywords with the strongest centrality are agriculture, ecology, growth, animal welfare, and diversity, but the centrality of the above keywords is less than 0.1, which indicates that scholars place differing emphasis on the study of biodiversity and human well-being. As a result, there are differences among keywords in the research results (when the centrality of a node is higher than 0.1, it can indicate that the node is a key node and is more representative [19]).

#### 3.5.2. Keyword Timeline Analysis

**Figure 8:**
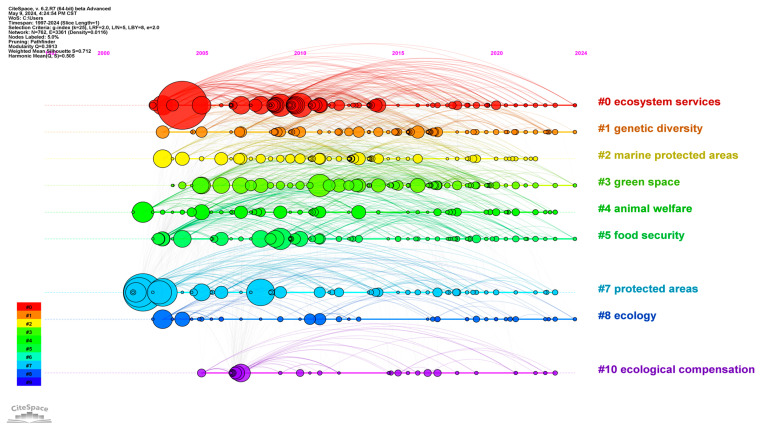
Keyword timeline graph from 1997 to 2024.

The analysis used the CiteSpace software, with the Log-Likelihood Ratio (LLR) as the calculation method, to cluster the keywords. The results showed that Modularity Q = 0.3913 > 0.3, and Silhouette = 0.712 > 0.5, indicating that the clustering structure is significant and the results are reasonable [13]. After clustering analysis, 16 clusters were obtained. By eliminating duplicate clustering words and clusters with less than 10 articles, a total of 9 clusters with the largest number of documents were obtained [16], as shown in Figure 8.

#0: ecosystem services mainly includes the following keywords: choice experiment, willingness to pay, human well-being, economic valuation, etc. Ecosystem services are the benefits that humans derive from ecosystems [20]. Since the 21st century, attention toward the “society–economy–nature” complex ecosystem has gradually increased [21,22]. This set of literature focuses on the relationship between ecosystem services and human well-being, as proposed in the “Millennium Ecosystem Assessment” by the United Nations General Assembly in 2001 [23,24]. It analyzes how to evaluate the economic, cultural [25,26], and other values of ecosystem services through choice experiment methods [27,28], and discusses residents’ and tourists’ willingness to pay for the restoration of ecosystem services [29,30]. Examining the relationship between ecosystem services and human well-being, in order to achieve a balance between the supply and demand of ecosystem services, is becoming an increasingly important aspect of biodiversity research for scholars in the global ecosystem services field.

#1: genetic diversity mainly includes the following keywords: gut microbiota, one health, disease, etc. This group of papers focuses on the microcosmic aspects of biodiversity that contribute to human well-being. Genetic diversity is an important prerequisite for the realization of biodiversity [31], and it is also a key factor for the survival of species and the normal function of ecosystems [32]. Existing studies have summarized the links between biodiversity, health, and disease, thus providing guidance for policy formulation by government departments [33,34]. In addition, some scholars have explored gut microbiota in depth, and proposed the importance of protecting gut microbiota for achieving biodiversity [35].

#2: marine protected areas mainly includes the following keywords: responses, risk, ecosystem-based management, competition, etc. Marine biodiversity conservation is an important part of biodiversity conservation [7,36]. This group of literature mainly focuses on marine biodiversity conservation, and marine ecological management can be effectively realized through marine spatial planning. Scholars have summarized the interaction mechanisms between human beings and marine ecosystems by exploring the impact of human activities under different management scenarios [37,38]. In addition, some scholars have further discussed the possible marine fishing competition among groups [39], assessed the risks and challenges it would bring to marine biodiversity conservation [40], and formed a plan to promote the sustainable development of marine protected areas [41]. Therefore, marine biodiversity conservation is also an important component of biodiversity conservation.

#3: green space mainly includes the following keywords: nature relatedness, mental health, urban planning, urban biodiversity, etc. The literature focuses mainly on urban biodiversity. Urban green space and urban biodiversity are closely related to human health and well-being [42]. The protection of urban biodiversity helps to coordinate public health relations and enhance the well-being of urban residents by protecting their mental health [43,44]. For example, natural correlation can significantly regulate this process; that is, the higher the natural correlation of urban residents, the more positive happiness they can show [45,46]. In addition, rational urban planning and growth management are conducive to promoting urban biodiversity conservation and enhancing ecosystem services [47]. Therefore, urban biodiversity conservation is also one of the main trends in current research.

#4: animal welfare mainly includes the following keywords: wildlife trade, environmental enrichment, animal behavior, etc. This group focuses on wildlife conservation. Due to rapid environmental change, wildlife is facing an increased risk of extinction [48]. In addition, the global wildlife trade is a growing threat to biodiversity, species conservation, and animal welfare [49,50]. In recent years, the widespread use of remote animal behavior monitoring technology has become conducive to supporting the formulation of management policies, such as animal welfare programs and animal remote protection, so as to improve the survival rate of wild animals and protect biodiversity [51].

#5 food security mainly includes the following keywords: productivity, agriculture, pest control, forest conservation, etc. Biodiversity conservation can solve the sustainability problems faced by modern intensive agriculture, and is an important way to achieve food security and sustainability [52]. Therefore, on the one hand, it is necessary to promote biological pest control and soil and water quality regulation [53], which is conducive to achieving the coordination of biodiversity and productivity development, thereby achieving global food security and minimizing environmental degradation [54]. On the other hand, promoting the sustainable development of forestry also plays a key role in biodiversity conservation and food security [55]. Through the construction of forest “granaries”, human food production can be increased, thus accelerating the construction of a diversified food supply system [56].

#7: protected areas mainly include the following keywords: climate change, biodiversity conservation, sustainable development, poverty, etc. This group of documents focuses on the construction of protected natural areas. Protected natural areas are one of the key means of biodiversity conservation [57], but are affected by climate change [58], which increases the risk of biodiversity loss [59]. In addition, protected areas are often located in generally poor areas, and the construction of protected areas can effectively prevent the decline of forest coverage, but at the same time reduce the income of farmers in the forestry industry [60]. Therefore, in order to further discuss the correlation between biodiversity conservation and residents’ income in poor areas, Ferraro proved through a series of studies that the construction of protected areas not only contributes to biodiversity conservation, but also improves residents’ income in poor areas [61].

#8 ecology mainly includes the following keywords: traditional, ecological, knowledge, stability, community structure, etc. The literature in this area focuses on the implications of traditional ecological knowledge for the study of ecology. Traditional ecological knowledge is a hot topic in the field of international biodiversity conservation [62,63]. Existing studies have repeatedly proven that the impact of community structure on the stability of biodiversity is consistent with prior knowledge [64].

#10: ecological compensation mainly includes the following keywords: expansion, global change, carbon cycle, etc. This group of literature mainly focuses on the role of ecological compensation in biodiversity conservation [65]. The literature explores the adverse factors that affect the global carbon cycle due to the encroachment of ecological land (such as forest, grassland, and wetland) by cultivated land expansion [66], and improves the method for calculating ecological compensation amount, thus reducing ecological footprint [67].

From the distribution years of the above nine literature clusters, it can be seen that relevant studies on biodiversity and human well-being paid more attention to the construction of protected areas and animal welfare issues earlier on. As the study of biodiversity and human well-being has received wider attention globally, relevant research on topics such as food security, ecosystem services, ecological compensation, and green space has gradually emerged and shown a trend of diversification.

#### 3.5.3. Keyword Burstiness Analysis

Keyword burstiness can show the keyword frequency in a specific period, in order to reflect the hot spots and frontier trends in a field [13]. Table 4 shows 25 mutational keywords from international studies on biodiversity and human well-being.

First of all, in terms of mutation intensity, the top five keywords are conservation (11.56), contingent valuation (10.66), ecology (10.08), economic valuation (9.02), and biodiversity conservation (8.31). In the corresponding period, these are important hotspots for research on biodiversity and residents’ well-being, which is consistent with changes in biodiversity conservation policies at home and abroad during the period. The keyword with the lowest intensity is opportunity (4.99), and its emergence lasted from 2017 to 2019. Secondly, from the perspective of mutation duration, the three keywords with the longest duration are environmental services, contingent valuation, and payments, which all lasted more than eight years. The keywords with the shortest duration are greenhouse gas emissions and wildlife trade, which lasted only one year.

Moreover, keywords such as parks, urban green space, and greenhouse gas emissions are the topics at the frontier of current research, and also the directions that need to be focused on in the future. Among these, urban green space and urban parks are some of the main components of sponge city construction, which has an important impact on greenhouse gas emission reduction [68]. Comprehensively and continuously promoting the construction of protected natural areas and urban green space can represent a realistic direction for future research on global biodiversity and human well-being.

Additionally, as China achieved its poverty alleviation goal in 2020, it can provide a model for other developing countries in the world. However, at the same time, as an important research site for the field of global biodiversity, China’s poverty alleviation has shown a decreasing trend in promoting global ecological poverty alleviation and poverty reduction. In order to further consolidate the achievements of poverty alleviation, how to prevent a large-scale return to poverty is still an important topic that global scholars must continue to explore for a long time in the future [69]. At the same time, in recent years, nature-based solutions (NbS) [70] and fragmentation [71] have gradually come to the forefront of the research in this field.

## 4. Discussion

The global research field of biodiversity and human well-being is constantly expanding, and a large number of research results have been accumulated so far, providing a solid foundation for future research. The authors of this study believe that future studies on biodiversity and human well-being should focus on the following four aspects:

(1) Enhance international cooperation and exchanges, and promote regional cooperation among research institutions. International cooperation is an important way to promote scientific development. Therefore, it is necessary to strengthen international cooperation in fields related to biodiversity conservation and the improvement of human well-being [72]. From an interdisciplinary perspective, encompassing social sciences, natural sciences, and medicine, efforts should be made to encourage scholars from different countries to better integrate into the study of biodiversity and human well-being [73]. The United Nations and governments of all countries should adopt joint tackling policies to encourage cross-border cooperation among experts and scholars in relevant fields. Starting with key issues such as talent and funding, we will realize the continuous implementation of cross-regional cooperation projects by scientific research institutions, and promote the high-quality development of theoretical and practical research in the field of global biodiversity and human well-being improvement.

(2) Build a framework for biodiversity system analysis driven by climate change factors. Climate change is an important influencing factor and one of the main threats to nature-led biodiversity change [74]. At present, the world is committed to promoting the process of climate governance and biodiversity conservation, focusing on greenhouse gas emissions [75], nature-based solutions [70], and other climate change impact factors, but it is still in its initial stage [76]. In the next step, we should build on the current thinking of climate change research, explore the integration of biodiversity and human well-being research into the “1 + N” policy system of carbon neutrality and carbon peaking, integrate naturalistic solutions (NbS) into the construction of ecological civilization, and explore global plans to reduce carbon and increase sink, to respond to climate change.

(3) Strengthen urban green space management and ecosystem diversity protection to improve residents’ happiness. Urban ecosystems support more than 70% of human activities. They are is comprehensive systems composed of the natural environment, social economy, and cultural science and technology. Furthermore, the conservation of urban ecosystem diversity is an inevitable requirement for maintaining global ecological security, providing ecosystem services, and supporting the well-being of urban residents [77]. Therefore, under the adverse impact of accelerating urbanization on ecosystem diversity, the construction and management of urban green spaces [42] and urban parks [78] will become increasingly important issues. In the future, a series of studies on the conservation of urban ecosystem diversity should be carried out from a multi-scale perspective. In addition, existing studies have shown that with an increase in biodiversity, people’s psychological and physiological benefits from urban green space can also increase [79]. Therefore, it is necessary to deepen research into the influencing factors of the conservation of urban ecosystem diversity on the improvement of residents’ happiness, and systematically comb out the formation mechanism of this process.

(4) Build a system of protected natural areas with national park groups as the main body, and prioritize the conservation of species diversity [80]. Habitat fragmentation is the main cause of biodiversity decline [81]. In recent years, the Chinese government has been consistently committed to improving its policy and regulatory system. Through the establishment of a natural protected area system with national parks as the main body, species diversity is being continuously enhanced and biodiversity protection is being strengthened [76]. National parks are a new type of natural protection system, in line with the inevitable trend of global ecological civilization construction [82]. Therefore, the next step is to deepen the spatial layout of national parks, functional zoning, marine national park construction, and ecological product value realization paths. Based on artificial intelligence and big data analysis, a national park monitoring system can be built to more accurately assess the effectiveness of species diversity conservation in national parks. In addition, it is necessary to further mediate the contradictions and cooperative relations among national park visitors, farmers, enterprises, governments, and other stakeholders, explore a new model of species diversity governance of national parks, and provide scientific guidance for government decision making.

(5) Integrate global gene bank resources to provide feasible solutions for genetic diversity conservation. Current research pays little attention to genetic diversity and the evolutionary continuum between populations and species [83]. However, with the increasing world population, the demand for genetic resources will rise. By protecting and maintaining genetic diversity, sustainable food security can be achieved [84]. Therefore, the next step is to integrate existing genomic data, and deeply explore the mysteries of species origins and evolution, thereby providing solutions for a series of major biological conservation issues and offering theoretical support for the protection of biodiversity [85].

## 5. Conclusions

Using Citespace 6.2.R7 software to draw a knowledge map, this study conducted quantitative statistical and visual analysis of 4453 international studies on biodiversity and human well-being from 1997 to 2024, taking into account the aspects of publication volume, discipline, major cooperative relationships, research path evolution, and frontier analysis. In summary, the following conclusions were reached.

First of all, in terms of the number of published papers and the subjects of the published papers, the number of published papers on biodiversity and human well-being has shown a steady upward trend, and has experienced an embryonic stage, a slow development stage, and a rapid growth stage from 1997 to 2024. At the same time, research on biodiversity and human well-being was wide-ranging, involving a total of 167 subject areas. In the fields of environmental science, ecology, and environmental research, the number of publications exceeded 900, indicating that the focus of research on biodiversity and human well-being is still the ecological environment. Furthermore, research related to biodiversity and human well-being has received a broad scope of funding support from various sources.

Secondly, from the perspective of research subjects and cooperation, the relevant research mainly involves 168 countries or regions, and the United States, England, Germany, Australia, and China are the main research countries in this respect. However, from the perspective of the composition of research institutions, CNRS in France, the Chinese Academy of Sciences in China, and INRAE in France are the main institutions in this field. Although the number of papers published by American scholars is large, the research institutions they belong to are scattered, and the University of California System in the United States ranks only fourth in the world.

Moreover, from the perspective of co-citation analysis, COSTANZA R., DÍAZ S., and STEFFAN-DEWENTER I. are the most-cited authors in the field of biodiversity and human well-being. In terms of journal citations, *Science* is the most cited journal, with 2145 citations, followed by *Nature*, *PNAS*, *Plos One*, and *Biological Conservation*.

Finally, from the perspective of research content and cutting-edge progress, ecosystem services, genetic diversity, marine protected areas, green space, animal welfare, food security, protected areas, and ecological compensation are the current hot topics in the study of biodiversity and human well-being. In addition, according to the results of keyword emergence, the research frontier currently focuses on parks, urban green spaces, greenhouse gas emissions, nature-based solutions (NbS), fragmentation, etc., indicating that climate change, urban biodiversity conservation, and national park construction are research directions worthy of focus in the future.

## 6. Limitations and Future Perspectives

This study has some limitations. Firstly, the data in this study are sourced solely from the Web of Science database; although this is one of the most recognized global databases, this means that research results published by Chinese scholars in databases such as CNKI have not been included. Therefore, in future research, we will conduct comparative analyses using different regional databases on biodiversity and human well-being. In addition, the study only used the CiteSpace software for analysis; in the future, mixed research could be conducted by combining literature visualization softwares, such as VOSviewer and Zotero.

## Figures and Tables

**Table 1:** Top 5 in funding.

No	Volume	Funding
1	30	National Natural Science Foundation of China (NSFC)
2	28	European Union (EU)
3	8	World Animal Protection
4	5	Natural Environment Research Council (NERC)
5	5	Swedish Environmental Protection Agency

**Table 2:** Top 10 institutions by publication volume from 1997 to 2024.

No	Volume	Centrality	Year	Institution	No	Volume	Centrality	Year	Institution
1	157	0.03	2005	Centre National de la Recherche Scientifique (CNRS)	6	99	0.04	2005	Wageningen University and Research
2	132	0.03	2006	Chinese Academy of Sciences	7	87	0.03	2004	University of London
3	106	0.07	2004	INRAE	8	80	0.06	2005	Consejo Superior de Investigaciones Cientificas (CSIC)
4	104	0.02	2006	University of California System	9	73	0.01	2012	University of Exeter
5	99	0.03	2008	Helmholtz Association	10	71	0.03	2003	CGIAR

**Table 3:** Top 10 journals by citations from 1997 to 2024.

No	Cited Times	Centrality	Year	Co-Cited Journal Name	No	Cited Times	Centrality	Year	Co-Cited Journal Name
1	2145	0.01	2002	*Science*	6	1432	0.04	2002	*Ecological Economics*
2	1848	0.00	2002	*Nature*	7	1370	0.01	2002	*Conservation Biology*
3	1838	0.02	2002	*PNAS*	8	1217	0.01	2002	*Bioscience*
4	1637	0.01	2010	*Plos One*	9	1136	0.02	2002	*Trends in Ecology and Evolution*
5	1471	0.03	2002	*Biological Conservation*	10	973	0.02	2005	*Journal of Environmental Management*

**Table 4:** Top 25 strongest burst keywords by citations in biodiversity and human well-being research from 1997 to 2024.

Keywords	Year	Strength	Beginning	End	1997–2024
ecology	2003	10.08	2003	2010	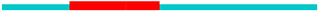
growth	2003	5.46	2003	2010	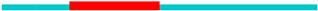
systems	2005	5.87	2005	2010	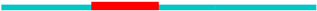
conservation	2002	11.56	2006	2013	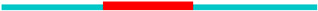
diversity	2002	5.12	2006	2008	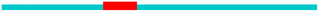
risk	2007	6.96	2007	2014	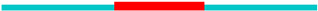
future	2007	6.61	2007	2013	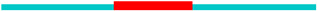
contingent valuation	2004	10.66	2009	2017	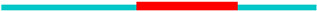
economic valuation	2010	9.02	2010	2015	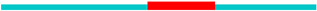
environmental services	2010	7.62	2010	2019	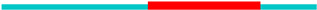
payments	2010	7.19	2010	2018	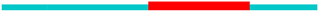
biodiversity conservation	2003	8.31	2011	2014	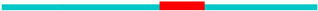
resources	2011	5.03	2011	2018	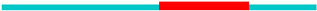
decision making	2013	6.45	2015	2020	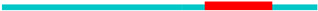
intensification	2015	5.97	2015	2018	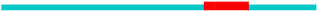
conceptual framework	2015	5.38	2015	2020	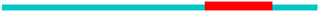
parks	2011	5.01	2015	2018	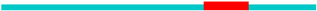
poverty alleviation	2016	5.71	2016	2019	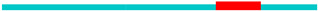
urban green space	2019	7.16	2019	2021	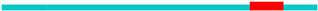
opportunity	2017	4.99	2019	2021	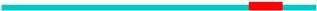
greenhouse gas emissions	2020	6.71	2020	2021	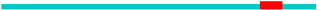
wildlife trade	2020	6.34	2020	2021	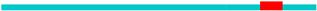
perspectives	2021	5.51	2021	2024	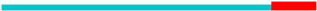
fragmentation	2021	5.20	2021	2024	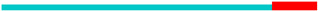
nature-based solutions	2017	5.02	2021	2024	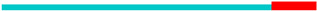

▃ Burst Time Period of Keywords. ▂ Years with No Significant Change in Keyword Frequency.

## Data Availability

The original contributions presented in this study are included in the article; further inquiries can be directed to the corresponding author.
